# Applicability and usefulness of pupillometry in the study of lexical access. A scoping review of primary research

**DOI:** 10.3389/fpsyg.2024.1372912

**Published:** 2024-03-11

**Authors:** Carlos Rojas, Yuri E. Vega-Rodríguez, Gabriel Lagos, María Gabriela Cabrera-Miguieles, Yasna Sandoval, Jaime Crisosto-Alarcón

**Affiliations:** ^1^Department of Health Rehabilitation Sciences, University of Bío-Bío, Chillán, Chile; ^2^Department of Spanish, University of Concepción, Concepción, Chile

**Keywords:** pupillometry, lexical access, word recognition, word retrieval, semantic processing

## Abstract

Pupil dilation has been associated with the effort required to perform various cognitive tasks. At the lexical level, some studies suggest that this neurophysiological measure would provide objective, real-time information during word processing and lexical access. However, due to the scarcity and incipient advancement of this line of research, its applicability, use, and sensitivity are not entirely clear. This scoping review aims to determine the applicability and usefulness of pupillometry in the study of lexical access by providing an up-to-date overview of research in this area. Following the PRISMA protocol, 16 articles were included in this review. The results show that pupillometry is a highly applicable, useful, and sensitive method for assessing lexical skills of word recognition, word retrieval, and semantic activation. Moreover, it easily fits into traditional research paradigms and methods in the field. Because it is a non-invasive, objective, and automated procedure, it can be applied to any population or age group. However, the emerging development of this specific area of research and the methodological diversity observed in the included studies do not yet allow for definitive conclusions in this area, which in turn does not allow for meta-analyses or fully conclusive statements about what the pupil response actually reflects when processing words. Standardized pupillary recording and analysis methods need to be defined to generate more accurate, replicable research designs with more reliable results to strengthen this line of research.

## 1 Introduction

Language processing is an expression of a complex cognitive function that undergoes significant changes throughout life (Marini and Andreetta, 2016). It develops rapidly in early childhood (Kuhl, 2010), remains stable in adulthood, and gradually declines with age (Peelle, 2019). During this cycle, lexical access occurs as a smooth and effortless process despite the different levels of processing involved (Goral et al., 2007; Shafto et al., 2010; Abrams and Davis, 2016). Lexical access allows us to accurately recognize (understand) or retrieve (produce) a word from the thousands in our vocabulary, allowing us to act effectively in the environment with the speed and accuracy that communicative exchange requires (Abrams and Farrell, 2011; James and Goring, 2018; Davis, 2020; Rojas et al., 2022). In this sense, lexical processing has become a frequent research focus in cognitive neuroscience over the last 40 years.

Lexical processing occupies a central place among language comprehension and production processes. It is a veritable meeting point between peripheral processes related to acoustic or visual perception (input signal) and speech or writing production (output signal). Moreover, it is associated with higher-order processes related to contextual meaning assignment, syntax, and discourse (Igoa, 2009; Abrams and Farrell, 2011; Rojas et al., 2022). On the other hand, lexical skills (i.e., activation-selection of lexical competitors and activation of core meanings) are described as an organized set of ‘subpersonal’ cognitive processes, which means that they are not under the voluntary control of the individual (Igoa, 2009; Cuetos et al., 2015). Generally, there is a consensus that lexical access can be divided into three levels of processing: (1) word recognition, (2) semantic activation, and (3) lexical retrieval.

First, at the word recognition level, a weighting (or comparison) is made between a physical stimulus from the environment (acoustic or visual signal) and a lexical representation stored in memory (Luce and Pisoni, 1998). This similarity weighting is performed between the input signal and a limited set of lexical competitors (recognition targets). Each competitor contains phonological, orthographic or morphological features similar to each other and the input signal (Igoa, 2009; Cuetos et al., 2015). Thus, the competitor that accumulates the highest activation level (due to its similarity to the input signal) is selected and suppresses the rest of the activated competitors (Balota and Chumbley, 1984).

On the other hand, the level of semantic activation involves the recognition of the semantic features of the selected lexical piece (i.e., features of shape, size, color, and other properties), the activation potential of which depends on variables such as the frequency of use of the word, its degree of imaginability, or the age of acquisition (Cuetos et al., 2015). Finally, the level of lexical retrieval (word production) begins with the activation of the concept that the speaker wishes to convey (Levelt, 1989). This process involves the activation of several competing lexical competitors (similar in form or meaning). Thus, the competitor that best represents the conceptualized idea is selected, and the rest are deleted (Igoa, 2009; Cuetos et al., 2015). Subsequently, the selected word is encoded at the syllabic, phonemic or graphemic level for its production.

In general, lexical access studies have very different objectives and can address any of the three levels of processing mentioned above (Perea and Rosa, 1999), which allows research ranging from the activation and selection of lexical competitors (recognition) to the selection of lemmas and subsequent phonological encoding (retrieval). Moreover, they incorporate all kinds of populations and age groups. For example, there are studies on the role of lexical access during language acquisition in early childhood (Dale and Fenson, 1996; Dromi, 1999) or studies on lexical availability in school and university students (Barbeiro et al., 2011; Richter et al., 2013; Ludewig et al., 2022). Many studies also document word retrieval difficulties in old age (Rojas et al., 2023) or verbal fluency difficulties in individuals with cognitive impairment or dementia (Henderson et al., 2023).

Various approaches are employed for its study (Perea and Rosa, 1999), including both traditional offline methods (chronometric tasks and psychometric tests) and online procedures using advanced instrumental techniques (e.g., electroencephalography/EEG, magnetic resonance imaging/MRI, and eye movements). In this context, it is worth mentioning that there has been a substantial increase in the utilization of pupillometry, particularly pupillary dilation, as a neurophysiological measure in cognitive language processing tasks at various levels over the past decade (Kuchinke et al., 2007; Goldinger and Papesh, 2012; Kuchinsky et al., 2013; Hepach, 2023).

Pupil size variation (dilations and contractions) is considered a basic neurophysiological indicator of central nervous system activity (Hyönä et al., 1995). However, research also suggests a connection between pupil size variation and the processing of complex cognitive information (Partala and Surakka, 2003; Liu et al., 2017; Ryals et al., 2021). The reason for this size pupil variation is that the sympathetic nervous system is activated during a complex cognitive task due to the effort put into solving it (Liu et al., 2017). Traditionally, the pupillary response has been thought to respond to modulation of the noradrenergic locus coeruleus (LC) in the brainstem, as its activity would release the norepinephrine (NE) necessary to produce generalized effects on central and peripheral nervous system function. As a result, physiological and cognitive activation would occur simultaneously (Samuels and Szabadi, 2008; Sara, 2009), generating a pupillary response evoked by the effort associated with the task being performed (Steinhauer et al., 2004; Liu et al., 2017).

However, recent studies suggest that pupillary responses are modulated by three brainstem neural systems (Joshi and Gold, 2020; Joshi, 2021), which in turn can be classified into different hierarchical levels of operation (Strauch et al., 2022). At the basic level, the pretectal olivary nucleus (PON), composed of parasympathetic and sympathetic circuits, would be responsible for controlling the pupillary reflex to light and focal distance (when fixating from far to near or vice versa; Joshi and Gold, 2020) through direct innervation of the pupillary muscles. On the other hand, the intermediate level of operation would consist of two partially overlapping neural circuits that are part of a global attentional network: (1) the locus coeruleus (LC)-centered circuit that generates pupil changes associated with attentional states (including pupil changes to outstanding stimuli); and (2) the superior colliculus (SC)-centered circuit that mediates pupil orienting responses (Strauch et al., 2022). Finally, at a higher hierarchical level, the locus coeruleus (LC)-norepinephrine (NE) neuromodulatory system would be responsible for generating responses to sensory control information, executive processing, and higher-level cognitive tasks (Strauch et al., 2022).

In this regard, the increase in pupillary diameter (or pupillary dilation) would be associated with the effort required for the cognitive task. For example, pupillary dilation is associated with the presentation of arousing stimuli or information, whether positive or negative, compared to neutral stimuli. Similarly, it is also connected to changes in cortical and subcortical activity related to cognitive behavior, such as attention lapses, learning of relevant information, and decision-making (Larsen and Waters, 2018; de Winter et al., 2021). Furthermore, pupillary dilation has been linked to processes of language acquisition and processing (Schmidtke, 2018), information storage, and retrieval (Eckstein et al., 2017).

Pupillary response has clear advantages over other cognitive measures, particularly behavioral measures. For example, it would provide information about the time (or precise timing) required for cognitive activation during highly complex tasks. Furthermore, the pupillary response could respond to cognitive processes that are partially activated but do not reach the level required for overt behavior or conscious awareness (Laeng et al., 2012). In addition, since it does not involve motor or verbal responses, it would prevent the conscious influence of executing these responses during processing. Therefore, pupillometry would be an appropriate method to assess cognitive processes during tasks that elicit high arousal, alertness, and emotional control (Ariel and Castel, 2014; Gomes et al., 2015; Kafkas and Montaldi, 2015; Larsen and Waters, 2018; de Winter et al., 2021).

In sum, it is possible to observe that many cognitive processes could cause pupil dilation (Laeng et al., 2012; Kafkas and Montaldi, 2015). In this sense, some studies note that pupil dilation could be an applicable and valuable tool for studying language processing (Schmidtke, 2018; Hepach, 2023). Pupillometry as a research paradigm in language and cognition has been used for quite some time but has only really taken off in the last decade. Linguistic-pupillometric studies have focused on three broad areas: (1) auditory and auditory–visual processing, (2) orthographic language processing, and (3) speech production. These have shown that pupillometry can provide researchers with new and creative ways to test hypotheses and thus advance knowledge of cognitive language processing (Schmidtke, 2018). However, it is not as well developed as other techniques used in this area, and there are still gaps regarding the meaning of this physiological response and its actual viability as a dependent variable in linguistic-cognitive research.

In this context, pupillometry could provide objective and real-time information about the cognitive effort or performance an individual experiences when processing words, for example, (1) when recognizing, inhibiting, and selecting words (McLaughlin et al., 2022), (2) when activating meanings (Chapman and Hallowell, 2015) or (3) during word retrieval and their phonological encoding (Granholm et al., 1998). In addition, pupillometry could reflect a person’s level of effort and surprise when the person must process ambiguous words, activate multiple meanings, or retrieve specific information (Pluchino et al., 2014). However, due to the limited and incipient advancement of this line of research in experimental psycholinguistics (in fact, most of the research developed is along the lines of auditory processing and speech production rather than lexical processing; Schmidtke, 2018), its applicability, use, and sensitivity are not entirely clear. Therefore, this scoping review aims to assess the applicability and usefulness of pupillometry in the study of lexical access.

### 1.1 Research questions

Based on the background presented so far, this scoping review formulates the following research questions: What is the applicability of pupillometry in the study of lexical access? What is the usefulness of this neurophysiological measure, and what are the specific aims of this area of research? What psycholinguistic methods, tasks, and procedures are used? Are the results of lexical-pupillometric research sensitive, reliable and replicable? What are the projections and limitations of this research area?

## 2 Methods

### 2.1 Context

Our approach to answering the research questions involved creating a protocol (Moher et al., 2015) that aligns with the objectives of a scoping review. This type of review aims to determine the scope and methodology of the literature on a specific topic and then provide a comprehensive summary of the available evidence (Munn et al., 2018). We developed eligibility criteria considering the research context and used critical concepts for the initial search phase. Finally, although the amount of research on lexical processing and pupillometry appears to be scarce, no grey literature was included in this review.

### 2.2 Search strategy and procedure

This scoping review was designed, conducted, and reported following the PRISMA Extension for Scoping Reviews (PRISMA-ScR) checklist and explanation (Tricco et al., 2018), and was supervised by one of the researchers (GL). The process included four stages: (1) search strategy definition, (2) literature screening, (3) data extraction, and (4) data synthesis and analysis.

#### 2.2.1 Search strategy

The search took place from November 25 to December 03, 2023, and included articles published in English from 2017 to date. The reasons for including articles since 2017 were: (1) before the search strategy phase, we refined and tested with different keywords, Boolean operators, and search years. As a result, we found that only a few articles before 2017 presented titles and objectives somewhat close to the purposes of the present review. However, we noted that these studies mainly focused on the role of pupillometry in acoustic speech perception and others on speech production, not necessarily on lexical access. Coincidentally, we also note that Schmidtke’s (2018) review, which focuses on language processing and pupillometry, is also dominated by auditory processing and speech production studies. Only a limited number of lexical-pupillometric studies from earlier years are mentioned, but they used acoustic stimuli as input signals (exclusion criterion for the present review, see Literature screening). (2) The inclusion of articles published in the last eight years allows us to make a very up-to-date first approach to lexico-pupillometric studies, identifying the essential research elements, recent gaps, future challenges, and current aspects that need to be better defined (essential objectives of a scoping review). However, beyond the decision to include articles from 2017 onwards, we cannot exclude the possibility that there are no lexico-pupillometric studies from previous years.

The search covered four databases: Web of Science (WoS), Science Direct, Scopus, and PubMed. The search strategy took a sensitive approach and included iterative processes using a combination of keywords, index terms, Boolean operators and search strings. The basic syntax and terms included were (((“Pupillometry” OR “Pupil dilation”) AND (“Words”))), which correspond to the natural language terms in this area of study (not included in MeSH) needed to generate the search strategy (Table 1). PubMed was used for the initial search, with subsequent adjustments for each database. The initial search was expanded to capture alternative words and phrases for each key term so that the search terms were tested and refined to ensure relevant and complete results. In this regard, “Lexical Access” was discarded and replaced with “Words” to avoid generalization during the identification phase. The literature was manually searched in databases and downloaded in a digital (.ris) format, then processed with the Rayyan platform.^1^

**Table 1:** Example of advanced search terms in Pubmed.

**Search:** ((pupillometry) OR (pupil dilation)) AND (words) (“pupillometry”[All Fields] OR (“pupil”[All Fields] AND “dilation”[All Fields]) OR “pupil dilation”[All Fields])) AND (“words”[All Fields] OR “worded”[All Fields] OR “wording”[All Fields] OR “wordings”[All Fields] OR “words”[All Fields]) **Translations** **pupillometry:** “pupillometry”[All Fields] **pupil dilation:** (“pupil”[All Fields] AND “dilation”[All Fields]) OR “pupil dilation”[All Fields] **words:** “word’s”[All Fields] OR “worded”[All Fields] OR “wording”[All Fields] OR “wordings”[All Fields] OR “words”[All Fields]

#### 2.2.2 Literature screening

All collected articles were reviewed by title and abstract. For this phase, two authors (CR and JC) utilized the Rayyan platform (a free AI web application; Ouzzani et al., 2016) to conduct the initial screening independently and autonomously using a double-blind function. Each reviewer labeled the identified articles in two categories: (1) included and (2) not included. In order to make this decision, the reviewers relied on the following inclusion criteria: (1) studies with an experimental, quasi-experimental, clinical trial, or cohort designs; (2) studies using pupillometry as a method of data collection; (3) studies examining changes in pupillary dilation associated with the following terms (tasks or levels): visual word recognition, word retrieval, lexical competitors, lexical activation, semantic activation or meaning activation; (4) studies in normal population or with cognitive impairments (congenital or acquired) and throughout the life cycle. The following exclusion criteria were used: (1) studies in pupillary dilation associated with the concepts: “speech recognition,” “auditory word comprehension,” “speech analysis,” “acoustic speech analysis,” or “speech perception”; (2) conferences, dissertations and theses.

The reasons for including articles with only visual (and not auditory) input were: (1) a large number of psycholinguistic studies with auditory stimuli focus on pupil responses in speech perception processes, phonetic-phonological signal analysis, acoustic analysis, and central auditory processing, levels that are not the focus of the present review; (2) the selection of only one type of sensory input allows greater control over the methodological and procedural characteristics of the included studies; (3) it also allows the analyzed pupillary response (results) to be more “homogeneous” when responding exclusively to visual lexical tasks; and (4) some hypotheses suggest that the sensory analysis of auditory stimuli presents problems of variability, segmentation and coarticulation, leading to greater use of cognitive resources. In contrast, the visual signal is relatively uniform and invariant, facilitating access to the lexicon and other higher cognitive processes.

Finally, if authors had conflicting decisions on an article, a third author (YEV) resolved the dispute and determined whether the article was included.

#### 2.2.3 Data extraction

Full-text versions of the selected articles were downloaded for analysis. Four authors independently reviewed the texts (CR, JC, YEV, MGC). Through discussion, the whole group resolved any discrepancies in the decision include/exclude any article. The final articles were chosen because they met the criterion of being consistent with the aim of the scoping review. Tables were designed in Word format to extract information on (1) objectives, (2) population and sample, (3) general characteristics of the assessed task, (4) method and design, (5) results, and (6) main conclusion. The primary reviewer (CR) confirmed that the included articles met the review’s purpose and inclusion/exclusion criteria previously controlled. Due to the varied methods, populations, and objectives of the studies included, meta-analysis was not the appropriate approach for this review. Finally, considering the scope of the topic and the purpose of a scoping review, no critical evaluation was performed on the sources of evidence included.

#### 2.2.4 Data synthesis and analysis

In order to present the results in a more systematic way, the included studies were categorized into three groups based on the levels of lexical processing reported in the literature. These groups were thus as follows: (1) studies on lexical recognition, (2) studies on semantic processing and activation, and (3) studies on lexical retrieval or word production. Finally, one of the authors (YS) was responsible for the review and consistency of the literature necessary for the introduction and subsequent discussion of the results.

## 3 Results

**Figure 1:**
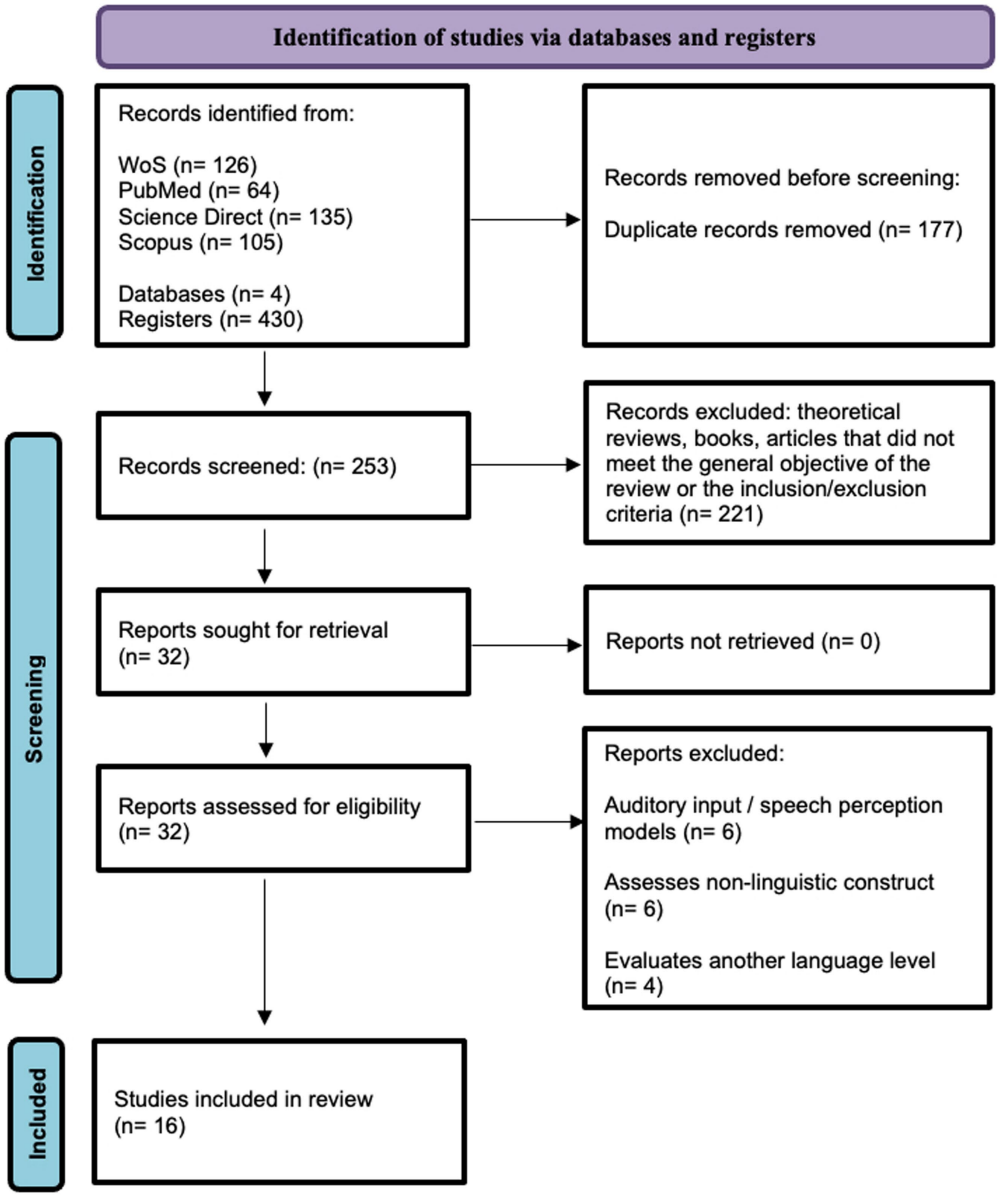
The flow chart of the screening process of identified and included studies.

The initial search identified 430 records. A total of 177 duplicate articles were removed. A total of 253 articles were reviewed, of which 221 were initially excluded (theoretical articles, articles that did not meet the purpose of the review, or inclusion/exclusion criteria). Thirty-two articles met the eligibility criteria, of which 16 were excluded (6 because they included auditory input in their methodology or assessed speech perception; 6 assessed non-linguistic constructs, and 4 assessed another linguistic level). The number of reports included in this review was 16 (Figure 1).

### 3.1 Studies on word recognition and pupillometry

Five of the 16 articles included in this review assessed the applicability and usefulness of pupillometry in visual word recognition (Table 2). In particular, 1 study (Haro et al., 2017) aimed to determine whether pupillary response is sensitive to lexical frequency and whether this effect is related to lexical access. Moreover, 2 articles focused on pupillary behavior during word recognition in bilinguals (Guasch et al., 2017; Toivo and Scheepers, 2019). Finally, 2 additional articles examined pupillary dilation (cognitive effort) in different types of readers during word recognition and subsequent reading (Shechter and Share, 2021; Shechter et al., 2022).

**Table 2:** Application and usefulness of pupillometry in lexical word recognition.

**Author**	**Title**	**Purpose**	**Sample size and population**	**General task characteristic**	**Method/Lexical task applied** (TDL, picture naming, priming, other)	**Results** (pupillary response to a lexical task)	**Main conclusion**
Haro et al. (2017)	Is pupillary response a reliable index of word recognition? Evidence from a delayed lexical decision task.	To test whether the modulation of the pupillary response by word frequency could be due to a confounding effect of response execution, or whether it rather reflects a genuine effect on word processing.	*N* = 60. Sixty Spanish speakers were recruited for the study. Half of them (21 women and 9 men; mean age = 20.63, SD = 3.18) participated in Experiment 1a, and the other half (28 women and 2 men; mean age = 19.70, SD = 2.52) participated in Experiment 1b.	Visual word recognition	In Experiment 1a, participants completed a standard LDT. In each trial a letter string representing a Spanish word or nonword. Participants were instructed to press the mouse button labeled “YES” or the one labeled “NO”, indicating whether or not the letter string was a word. In Experiment 1b, participants were asked to perform a delayed LDT. Unlike Experiment 1a, in this case the string of letters remained on the screen for 500 ms and was followed by a new fixation cross for 1,500 ms. Next, a question appeared on the screen (press button YES/NO). Pupil size was recorded with an EyeLink 1,000.	The study showed that the pupillary response was modulated by the lexical frequency of words in both experiments. Specifically, pupillary dilation increased when participants recognized lower frequency words than when they were confronted with higher frequency words, for both standard and delayed LDT tasks (*p*-values <0.05).	The results showed that pupillary response was modulated by word frequency in both the standard and the delayed LDT. Therefore, the present study provides evidence of the reliability of pupillometry for word recognition research. In addition, the pupillary response represents a purer measure of word processing than do behavioral responses (e.g., RTs or percentages of errors), given that the latter do not allow us to separate the processing and response components in the LDT.
Guasch et al. (2017)	Pupil dilation is sensitive to the cognate status of words: further evidence for non-selectivity in bilingual lexical access.	To replicate the cognate facilitation effect by examining reaction times and pupil responses.	*N* = 35. Thirty-five Psychology students (28 women), age ranged from 18 to 40 years old (Mage = 21.94, SD = 4.87). None of them reported a history of general impairment. Participants were all native speakers of Catalan and Spanish.	Visual word recognition	75 words and 75 pseudowords were selected for a standard LDT. The words were divided into three experimental conditions according to the degree of formal overlap between the Spanish words and their Catalan translations. The three conditions were: identical cognates (i.e., reina, reina in both Spanish and Catalan; “queen”), non-identical cognates (i.e., escuela, escola in Spanish and Catalan, respectively; “school”), and non-cognates (i.e., hacha, destral in Spanish and Catalan, respectively; “axe”). Participants were instructed to press with the right hand either the mouse button labeled as “YES” (left button) or “NO” (right button), as quickly and accurately as they could, indicating whether or not the letter string was a Spanish word.	Pupil response revealed that peak latency did not differ across experimental conditions. However, significant differences were observed when examining peak pupillary dilation. Pairwise comparisons showed that non-cognates elicited greater pupillary dilations than cognates (*p* = 0.01). On the other hand, there was no significant difference between identical and non-identical cognates, nor between non-identical and non-identical cognates.	The study add to past research in bilinguals showing that pupil response is sensitive to particular characteristics of word. In this sense, pupil response is also sensitive to cognate status. This result supports the potential utility of pupillometry in bilingualism research, a technique that has several advantages, such as that it is difficult to be controlled voluntarily and that it provides information about the time course of processing difficulty in a noninvasive way.
Toivo and Scheepers (2019)	Pupillary responses to affective words in bilinguals’ first versus second language.	To test whether words associated with high emotional arousal evoke greater pupil dilation than words associated with low emotional arousal. Assuming a reduced emotional response in L2, a greater affective pupillary response is expected for L1.	*N* = 96 Participants were aged between 17 and 33 years (mean age: 22 years). Were incorporated 32 English monolinguals, 32 Finnish-English bilinguals and 32 German-English bilinguals.	Visual word recognition	90 word-stimuli (30 high arousal, 30 low arousal, and 30 filler) were presented. At the end of each trial, a blue question mark asked participants to make a word recognition judgment, i.e., to press the right trigger (for “yes, I recognized the word and its meaning”) or the left trigger (for “no, I did not recognize the word or its meaning”) of a response device. Pupillary dilation was recorded with SR-Research EyeLink II.	For the German and Finnish bilingual groups, a significant effect of word type on pupillary responses was found only when the stimuli were presented in their respective L1 (*p* < 0.05). When the same participants were tested in L2 (English) there was no reliable effect of word type on pupil size (*p* < 0.05).	These findings showed that there is a reduced emotional resonance in L2 based on the pupillary responses and indicate that pupillometry is a promising alternative to skin-conductance research when measuring direct physiological responses to emotional content in different languages.
Shechter and Share (2021)	Keeping an Eye on Effort: A Pupillometric Investigation of Effort and Effortlessness in Visual Word Recognition.	To examine the cognitive effort during visual word recognition (reading) between expert adult readers and primary school pupils using pupillometry.	*N* = 98. Exp1-2 (*N* = 50): University students. Without reading difficulties, normal or corrected vision. Exp 3 and 4 (*N* = 48): Fourth to sixth graders. Without reading difficulties, normal or corrected vision.	Visual word recognition and oral and silent reading	Exp1-2: University students had to oral (exp1)/silent (exp2) read words (80), pseudowords (80) and fillers (80) Exp3-4-: School students had to oral (exp3)/silent (exp4) read words (80), pseudowords (80) and fillers (40). All tasks were presented in computer and the Pupil dilation was evaluated using Eyelink 1,000 Plus.	Relative to changes in pupil, experiments 1–4 showed a significantly main effects for word familiarity (*p* < 0.05). Specifically, larger pupil size dilation for pseudowords (especially if the string of letters was longer) than for real words in all tasks.	Pupillary responses are sensitive to the cognitive effort involved in single-word reading not only among skilled readers but also among school-age readers in both oral-and silent-reading modes. Pupillometry may offer a more sensitive moment-by-moment glimpse into the dynamics of word recognition (including developmental, interindividual, and intraindividual variation) that goes beyond the standard measures.
Shechter et al. (2022)	A pupillometric study of developmental and individual differences in cognitive effort in visual word recognition.	To measure and compare cognitive effort as an indicator of cognitive efficiency, using pupillometry to examine differences in word reading among adults and children.	*N* = 68 34 university students (27 females; mean age 27) and 34 the school-aged sample (19 females; mean age 10) from the fourth to the sixth grades.	Visual word recognition and reading aloud words and pseudowords.	Participants were asked to read aloud the displayed word which disappeared automatically. The trial sequence was the same for the adults and children except for a longer duration of stimulus presentation for the younger sample. Pupillary dilation was recorded during task execution with an EyeLink 1,000 Plus.	Children invested more effort in reading than adults, as indicated by larger and sustained pupillary responses (*p* < 0.001). In addition, while the fastest readers and the slowest readers invested a similar degree of overall cognitive effort in word reading, the fastest readers demonstrated accelerated pupillary responses compared to the slowest readers (*p* < 0.001).	Findings provided evidence for the existence of developmental differences in cognitive efficiency between readers in accordance with their reading proficiency. Quantitatively, skilled adult readers showed lower levels of cognitive effort when reading a word compared to school-aged readers. Qualitatively, their superior word recognition skill was manifested in differences in the temporal pattern of changes in pupil dilation over time as well.

In the first study, Haro et al. (2017) assessed pupillary response in a traditional lexical decision task (LDT, experiment 1) and a delayed lexical decision task (LDT delayed, experiment 2). During the experiments, 60 participants (30 per group) had to discriminate between pseudowords and words while their pupillary response was monitored. The words had different lexical frequencies (low, medium, or high). The results of the study suggest that pupillary response is a sensitive indicator of word frequency, with low-frequency words causing significantly grater pupil dilation than high-frequency words, regardless of the LDT type. The authors argue that pupil dilation would represent a genuine effect of lexical processing and could be considered an objective measure for assessing word recognition, as it would not be mediated by response mechanisms accessory to LDT (i.e., executive control, inhibition or sensorimotor aspects).

Among the articles focusing on bilingualism, Guasch et al. (2017) studied how bilinguals’ pupillary response is affected by words that fulfill the cognate condition, i.e., words from different languages with similar spelling and identical meaning. Using LDT, pupil dilation was recorded in 35 bilingual native speakers (Spanish-Catalan) when they processed words in the identical cognate/non-identical cognate/non-cognate condition. The study showed that the non-cognate word condition (words with no spelling overlap but identical meaning) produced a significantly greater pupil dilation than the other experimental conditions. The research provided evidence that pupillometry is sensitive to specific word characteristics (semantic-orthographic congruence/incongruence) in bilingual native speakers. Along the same lines, Toivo and Scheepers (2019) assessed the sensitivity of the pupillary responses to words of high emotional arousal words in L1 and L2 using LDT. They included 96 monolingual and bilingual young adult participants. The study revealed that emotionally loaded words in the native language (L1) produced a stronger pupillary response (increased dilation) than emotionally loaded words in L2, possibly due to the richer semantic representation created by emotional words in L1. According to the authors, pupillometry could be an objective and valuable tool for assessing the recognition of emotionally loaded words.

Finally, 2 articles were compiled that examined the applicability and usefulness of using pupillometry as an indicator of cognitive effort in word recognition in diverse type of readers. In example, Shechter and Share (2021) examined the pupillary response during a task with known words and pseudowords (letter strings of different lengths) in 48 university students and 48 schoolchildren. Consequently, longer pseudowords resulted in greater pupillary dilation, indicating increased cognitive effort of both experienced readers (university students) and novice readers (schoolchildren), both for oral and silent reading task. The authors project that pupillometry may offer a more sensitive moment-by-moment glimpse into the dynamics of word recognition (including developmental, interindividual, and intraindividual variation). In a second study, Shechter et al. (2022) assessed the sensitivity of the pupillary response during recognition and reading aloud of a series of words in children (*N* = 34) and young adults (*N* = 34), who were further classified into fast/slow readers. The results showed that children showed more significant cognitive effort (as evidenced by a greater pupillary response) than adults during word recognition and reading aloud. However, no differences in cognitive effort were observed between fast and slow readers in any age group. According to the authors, the pupillometric index would offer objective hints about how reading ability develops starting in the earliest phases of life.

### 3.2 Studies on activation and semantic word processing and pupillometry

Seven of the 16 articles reviewed (Table 3) evaluated the applicability and usefulness of pupillometry in activation and semantic word-processing tasks. One article relied on embodiment theory to show whether there is a significant pupillary response when processing words whose meaning is associated with a higher or lower level of brightness (Mathôt et al., 2017). Five additional articles examined the behavior of the pupillary response when establishing congruence or association judgments between pairs of words with specific semantic and orthographic features (Geller et al., 2019; Egan et al., 2020; Reilly et al., 2020; Egan et al., 2023; Haro et al., 2023). Another study (1) examined pupillary response sensitivity to determine whether the semantic priming effect is present in children under 24 months of age (Angulo-Chavira and Arias-Trejo, 2021).

**Table 3:** Application and usefulness of pupillometry in lexical semantic word processing.

**Author**	**Title**	**Purpose**	**Sample size and population**	**General task characteristic**	**Method/Lexical task applied** (TDL, picture naming, priming, other)	**Results** (pupillary response to a lexical task)	**Main conclusion**
Mathôt et al. (2017)	Pupillary Responses to Words That Convey a Sense of Brightness or Darkness.	To show that word meaning by itself can trigger a pupillary light response, an involuntary movement that has traditionally been believed to be a low-level reflex to light.	*N* = 90. Ninety naive observers (age range: 18–54 years) participated in the visual, auditory and control experiment, although only 30 participated in the visual recognition experiment.	Semantic Processing in the context of embodied language theory	For the main experiments, the semantic brightness of the words was varied. There were four categories of words: words conveying brightness (*n* = 33), words conveying darkness (*n* = 33), neutral words (*n* = 35), and animal names (*n* = 20). The participants’ task was to press the space bar whenever they saw an animal name and to withhold response otherwise. Word order was fully randomized. Pupil size was recorded monocularly with an EyeLink 1,000.	As predicted, pupils were smaller (*p* < 0.05) when participants read or heard words conveying brightness compared with words conveying darkness.	Eyes’ pupils are smaller after people read or listen to words conveying brightness (e.g., sun) than when people read or listen to words conveying darkness (e.g., night). This effect arises slowly and gradually and, in these experiments, peaked between 1 and 2 s after word onset. Therefore, the word meaning is sufficient to trigger a pupillary response, even when this response is not imposed by the experimental task, and even when this response is beyond voluntary control.
Egan et al. (2020)	How alliteration enhances conceptual attentional interactions in reading.	To characterize how the dynamic interactions between the phonological form and meaning of words can explain the sound relations between words in literary genre.	*N* = 20. Native English with normal or corrected-to-normal vision and no past or present diagnosis of a learning difficulty (16 females, mean age 22, SD 2.97).	Semantic processing (congruency) and form repetition (alliteration)	Participants were required to read adjective-noun word pairs in four conditions: congruent/alliterative (dazzling diamond), congruent/non alliterative (sparkling diamond), incongruent/alliterative (dangerous diamond), and incongruent/non alliterative (spooky diamond). The effects were assessed by Event Related Potential (ERP) and pupillary dilation. The participant had to indicate, by pressing a counterbalanced binary decision button, whether or not the two words were related in meaning. Pupil size was recorded in both experiments with an EyeLink 1,000 (EyeLink 1,000).	Congruency significantly modulated pupil dilation from 980 to 2000 ms, manifesting as a pupil size increase for congruent (alliterative/no alliterative) relative to incongruent (alliterative/no alliterative) word pairs (*p* < 0.05). In addition, alliteration and semantic relatedness interact such that pupil dilation increase is particularly sustained for related words within a phrase.	Bearing in mind that the course of pupil dilation manifests as a biphasic pattern, reflecting partially separable processes, the results suggest that semantically congruent pairs elicited greater autonomic arousal compared with incongruent pairs. ERP and pupil dilation data suggest that alliteration modulates online semantic processing. Thus, alliteration strategically arouses attention during reading and when comprehension is challenged, phonological information helps readers link concepts beyond the level of literal semantics.
Geller et al. (2019)	A Pupillometric Examination of Cognitive Control in Taxonomic and Thematic Semantic Memory.	To tested cognitive control requirements of retrieving taxonomic and thematic knowledge using a physiological measure of cognitive effort: pupil dilation.	*N* = 60. Sixty university students. All of the participants reported normal/corrected-to-normal vision and hearing, and reported no history of motor, cognitive, or neurological disorders.	Semantic relatedness judgement task	Participants were to respond to a semantic relation judgment task. Each participant completed 128 trials in a 2 (semantic type: Taxonomic vs. Thematic) × 2 (semantic strength: High vs. Low) factorial design. Were instructed to judge pairs of words as related or unrelated in meaning, they responded by pressing either the left button (“related”) or the right button (“unrelated”) on the gamepad. Pupil data were continuously acquired monocularly using a remote EyeLink 1,000 plus eye tracker.	The results showed that they did not differ significantly between taxonomic and thematic relationships. However, there was a greater pupillary response for the weak-relation than the strong-relation pairs, particularly the taxonomic types in the different time windows (*p* < 0.05), reflecting continued cognitive effort. In contrast, for low/high relatedness thematic pairs, pupil size did not increase substantially.	Detecting taxonomic relationships resulted in longer reaction times and a steeper pupil dilation slope than detecting thematic relationships. For relatedness strength, behavioral and pupillometric analyses revealed that weak relations were more difficult than strong relations. Critically, pupillometric data indicate that semantic control demands are primarily determined by relatedness strength, not whether the relationship is taxonomic or thematic.
Reilly et al. (2020)	Neuromodulation of cursing in American English: A combined tDCS and pupillometry study.	To determine whether right vs. left lateralized prefrontal neurostimulation via transcranial direct current stimulation could modulate taboo word production in neurotypical adults.	*N* = not informed. Participants included young adults (18–35) distributed in two groups: tDCS Condition A and tDCS Condition B. Each group will receive anodal stimulation or cathodal stimulation.	Activation and Semantic Processing	The study contrasted reading latencies and pupillary response patterns as functions of word type (taboo/non-taboo), polarity (cathodal/anodal), and time (pre/post stimulation). In the pre-stimulation condition, participants read aloud lists of taboo/non-taboo words. Participants were then subjected to a 20-min intervening session of neurostimulation. In the post-stimulation session, participants read aloud lists of taboo/non-taboo words matched in form to the pre-stimulus list. Pupil size was recorded in both experiments with an EyeLink 1,000.	Pupillary responses demonstrated a crossover reaction, suggestive of modulation of phasic arousal during cursing. Participants in the right anodal condition showed elevated pupil responses for taboo words post stimulation (*p* < 0.05). In contrast, participants in the right cathodal condition showed relative dampening of pupil responses for taboo words post stimulation.	These findings as supporting modulation of right hemisphere affective arousal that disproportionately impacts taboo word processing. The pupillary reaction observed in this study is explained by the fact that the pupil dilates parametrically in response to affective arousal, both for negative and positive. Second, the pupil dilates as a function of inhibitory control in tasks that require conscious suppression of a prepotent response. In this case, cursing behaviors probably load heavily on both affective arousal and inhibitory control. The contributions of valence and inhibitory control remain unclear.
Angulo-Chavira and Arias-Trejo (2021)	Mediated semantic priming interference in toddlers as seen through pupil dynamics.	To explore whether 24-month-old toddlers show within-level semantic mediated priming effects (e.g., cat [prime] – mouse [mediator] – cheese [target]) evaluated with a preferential looking and pupil dilation.	*N* = 27. Toddlers with a mean age of 24.1 months (SD = 0.34, range = 23.5–24.7). All toddlers were born at full term, were monolingual Mexican Spanish speakers, and had no hearing or visual problems.	Semantic/spreading activation between semantic levels (how meanings and their associations are organized in the lexicon)	Toddlers observed 8 experimental trials set up on a computer. The experimental trials were divided into two conditions: the related condition (the prime and target words were indirectly related at the semantic level through the mediating word) and the unrelated condition (the prime and target words were neither semantically nor associatively related). Preferential gaze and pupillary dilation were assessed using Tobii TX300.	Pupil dilation was measured from 150 to 1800 ms. Pupil dilation was greater in related trials than in unrelated trials from 1,188 to 1,212 ms relative to the target onset (cluster = 16.05, maxt = 2.01, *p* = 0.005) and from 1,322 to 1,688 ms (cluster = 236.09, max-*t* = 2.23, *p* < 0.001).	The results of gaze and pupillary dilation confirm that 24-month-olds show mediated priming, but an inhibitory mechanism acts to limit the spread of activation. This inhibitory mechanism may be slow and cognitively demanding and seems to operate at the semantic level or at least in the taxonomic-associative relationships between words.
Haro et al. (2023)	Pupillometric and behavioral evidence shows no differences between polyseme and homonym processing.	To examine whether differences between polysemes and homonyms appear, or become more pronounced, in tasks that involve increased semantic processing compared to less semantically engaging tasks, and to test if such differences are reflected in pupillary responses.	*N* = 117. 40 Spanish speakers took part in Experiment 1. 25 Spanish speakers took part in Experiment 2. 28 Spanish speakers took part in Experiment 3. 24 Spanish speakers took part in Experiment 4.	Semantic categorization (Experiment 3) and number-of-meaning task (Experiment 4).	Experiment 1 (LDT) was only used to standardize the material to be used. In Experiment 2, pupillary responses were recorded during a standard LDT. In Experiment 3, the participants completed an semantic categorization task (i.e., “Does the word belong to the category jobs, professions, and ranks?”), during which the researchers collected pupillary data. In Experiment 4, pupillary responses were recorded in a task where participants had to indicate whether the words had one or more meanings. The diameter pupil was recorded using an EyeLink 1,000 eye tracker.	In experiment 3 pupillary response is sensitive to word properties, as category-congruent words showed greater pupillary dilation than category-incongruent words (BF10 > 30). Cognitively it was more demanding to check that a word that referred to “jobs, professions and ranks” belonged to this category, than to check that a word that did not refer to this category did not belong to it. In Experiment 4, a clear ambiguity effect (higher pupillary dilation) was observed, reflected in participants activating different meanings for ambiguous words relative to unambiguous words (BF10 > 30). Differences between polysemes and homonyms were not observed in any task.	A first approach to the study of the pupillary response in the processing of lexically ambiguous words is provided. Specifically, a larger pupil dilation was observed for ambiguous words in comparison to unambiguous ones in number-of-meanings task (this higher cognitive cost of ambiguous words was reflected in the increased pupillary response). However, differences between polysemes and homonyms were not observed in any task. These results provide no evidence that polysemes and homonyms are processed differently.
Egan et al. (2023)	The impact of phonological relatedness on semantic congruency judgements in readers with dyslexia: Evidence from behavioral judgements, event related potentials and pupillometry.	Examine the potential for phonological information to differentially modulate the semantic congruency effect in dyslexic and typical reader groups.	*N* = 38. Fifty-four native English speakers were originally recruited (16 were discarded), comprising 19 typical readers and 19 readers with developmental dyslexia.	Recognition. Semantic judgments	Participants saw adjective-noun word pairs orthogonally manipulated for semantic congruency and alliteration (104 pairs per condition). The adjective was then presented for a random duration in the range of 330–550 ms in 20 ms increments. In half of the experimental trials, the noun was then presented for 500–600 ms in random 20 ms increments. In the other half, the noun was presented for 2000 ms. A response cue then prompted the participant to indicate, button press, whether the two words were related in meaning. Pupillary dilation was recorded with EyeLink 1,000.	An early main effect of alliteration was present from 70 to 350 ms, showing overall larger pupil dilation to alliterating pairs. A main congruency effect was seen from 1,270 to 2000 ms, characterized by consistently larger pupil dilation to congruent versus incongruent trials. From 1,350 to 2000 ms, a main effect of group was seen such that dyslexic participants showed generally reduced pupil dilation compared to typicals.	Dyslexic readers showed smaller pupillary dilation overall than typical readers during the last dilation phase. It is proposed that the attentional system of dyslexic readers may be less engaged with these word stimuli than is the case for typical readers, i.e., dyslexic readers appear to produce less autonomic arousal in response to print than typical readers.

In the first study reviewed, Mathôt et al. (2017) assessed pupillary response in a semantic recognition task within the framework of embodied language theory. In the experiment, participants (30 in total) were tasked with identifying words that were semantically linked to brightness, darkness, neutrality (words that did not evoke brightness or darkness), as well as animals (words whose meaning is not associated with any aspect of brightness or darkness). Participants were instructed to press the space bar when animal-related words appeared on the screen, while their pupillary response was monitored throughout the trials. The pupillary response, which reacts to changes in illumination, showed a smaller dilation when words linked to brightness were activated but a larger dilation when processing words related to darkness. The authors confirm that the pupillary response is consistent with embodied language theory, as semantic activation of word meaning was sufficient to elicit a response without needing an external light stimulus.

In the case of articles focusing on pupillary response to judgments of congruence or semantic relatedness between word pairs, Egan et al. (2020) used Event-Related Potential (ERP) and pupillometry to examine how alliteration (similar phonological form) between words improves attentional interactions during reading. Twenty adult participants were asked to make semantic judgments between semantically congruent/incongruent word pairs that also satisfied the alliteration/non-alliteration condition. The results showed that the pupil response was larger for word pairs that met the congruence condition (alliterative or non-alliterative). The research also found that the interaction between alliteration and congruence led to a continuous increase in pupil dilation when related words appeared in a sentence. According to the authors, alliteration, as measured by ERP and pupillometry, can influence semantic processing by supporting concept activation during comprehension errors. The same authors (Egan et al., 2023) evaluated the sensitivity of pupillometry to measure the influence of phonological relatedness on semantic congruence judgments in readers with dyslexia. 38 participants with (19) and without dyslexia (19) were asked to semantically process orthogonally manipulated word pairs for semantic congruence and alliteration (phonological form). Participants had to indicate whether the two words presented were meaning-related by pressing a button. As a result, pupil dilation was consistently lower in dyslexic readers. According to the authors, people with dyslexia show reduced engagement of attentional processes during reading, making pupillary response a sensitive measure for detecting differences in lexical processing among readers.

Geller et al. (2019) examined the utility of the pupillary response in a processing task of taxonomically or thematically semantically related word pairs, whose relatedness could be weak/strong. Sixty undergraduates had to make judgments about the relatedness/unrelatedness of the word pairs. The results showed greater pupil dilation in weakly related pairs than in strongly related pairs (particularly taxonomic pairs), where greater pupil dilation would require greater cognitive effort. The pupillary data would suggest that semantic control demands would be determined primarily by the strength of the semantic relationship (weak/strong) and not necessarily by the type of relationship (taxonomic or thematic). In a similar study, Haro et al. (2023) examined whether the pupillary response was sensitive to differences between polysemous and homonymous words when performing tasks involving different processing costs. The authors conducted four experiments on a sample of 117 adult subjects. Specifically, in Experiment 3 (*n* = 28), participants had to solve a semantic categorization task, and in Experiment 4 (*n* = 24) they had to determine whether a word had more than one meaning. Greater pupil dilation was only observed for those words that were congruent to the semantic category evaluated (Exp.3) and when ambiguous words were processed (Exp.4). No effects were observed when comparing polysemous and homonymous conditions. The authors point out that there is insufficient evidence to demonstrate differences in the processing of polysemous/homonymous words. However, the processing of ambiguous words and those that are of the same semantic category, would generate greater cognitive cost and therefore greater pupillary response.

In the same line, but with different aims, Reilly et al. (2020) employed pupillometry to assess the impact of transcranial stimulation on the processing of taboo and non-taboo words. Adult participants were required to read aloud words of taboo/non-taboo meaning before and after transcranial stimulation. As a result, transcranial stimulation on the right cerebral hemisphere impacts taboo word processing, which is reflected in increased pupillary dilation. In their conclusion, the authors state that the pupillary response to taboo words after transcranial stimulation results from the activation of emotional valence and the person’s unconscious inhibitory control when processing socially inappropriate words. Finally, Angulo-Chavira and Arias-Trejo (2021) explored the pupillary response when executing a semantically mediated priming task within the same level (i.e., cat [prime] - mouse [mediator] - cheese [target]) to determine whether this semantic effect is present in children younger than 24 months. Twenty-seven children observed eight experimental trials set up on a computer. The trials were divided into two conditions: the related condition (target and cue words had an indirect relation) and the unrelated condition (target and cue words had no semantic or associative relation). The pupillary response shows that children present mediated priming effects from 24 months onwards. However, the authors argue that there may be inhibitory mechanisms that limit their activation from an early age.

### 3.3 Studies on word retrieval and pupillometry

Four of the 16 articles found (Table 4) evaluated the applicability and usefulness of pupillometry during word retrieval. Three articles address pupillary response in verbal fluency tasks (multiple word retrieval) in various populations, and the remaining one addresses pupillary behavior in the presence of the tip-of-the-tongue states (TOTs) in adults.

**Table 4:** Application and usefulness of pupillometry in lexical word-retrieval.

**Author**	**Title**	**Purpose**	**Sample size and population**	**General task characteristic**	**Method/Lexical task applied** (TDL, picture naming, priming, other)	**Results** (pupillary response to a lexical task)	**Main conclusion**
El Haj et al. (2020)	Pupil dilation as an index of verbal fluency.	To determine pupillary behavior during the application of a phonological verbal fluency task.	*N* = 45. Graduate and undergraduate students (25 women, 20 men. Range = 23.55 years, SD = 5.32). French Monolingual, sensory, cognitive and psychiatric immunity.	Word retrieval/lexical-phonological and inhibitory retrieval processes	Participants had to perform two task. First a phonological verbal fluency (words that begin with the letter “p” for 1 min). Second, they had to perform the verbal task of counting numbers out loud. Participants had to look at a black cross drawn in the center of a paper while performing the task. Pupil dilation was evaluated using Eye-Tracking glasses.	Larger pupil diameter was observed during the verbal fluency task (*M* = 3.07, SD = 0.86) than during counting (*M* = 2.60, SD = 0.88) [*t*(44) = 4.22, *p* < 0.001, Cohen’s *d* = 0.59]. Regardless. the participants generated a mean of 20.55 words (SD = 5.62).	The larger pupil dilation during verbal fluency than during counting can be attributed to the cognitive (i.e., executive) load of the verbal fluency task. This task required participants to evaluate retrieved information, inhibit inappropriate information, and search for an alternative. Pupillary dilation could be used as a physiological marker of verbal performance.
Ryals et al. (2021)	Increased pupil dilation during tip-of-the-tongue states (TOTs).	To determine if there is an increase in pupillary dilation during the presence of TOTs events, as a result of the cognitive effort generated by such events.	N = 46. Undergraduate students (26 women, 20 men). Age 18–45. Normal vision o corrected.	Word retrieval/lexical-phonological and inhibitory retrieval processes	Participants completed 100 trials of questions (Nelson and Narens general knowledge norms). Questions were presented for 4 s which they freely scanned the word. They were then asked to attempt to mentally retrieve an answer for 4 s, during which time pupil dilation was recorded (EyeLink 1,000 plus). Participants were then asked to indicate whether they were experiencing a TOT state according to specific classification.	A paired samples *t*-test revealed that, when unable to retrieve a question’s answer, participants exhibited a significantly higher mean pupil area among reported TOT states than reported non-TOT states [*t*(45) = 8.08, *p* < 0.00001, Cohen’s *d* = 1.18], 95% Confidence Interval of the Difference [88.65, 147.52].	The increased pupil dilation during TOTs is consistent with the notion that TOTs are accompanied by feelings of arousal and excitement and that they involve curiosity and information seeking. TOTs are a form of retrieval success rather than failure. TOT could represent access to partial target information, and the increase in pupil diameter found in the present study reflects the amount of information retrieved.
El Haj et al. (2022)	The talking eyes: Pupillometry to index verbal fluency in normal aging.	Investigated whether pupillometry may assess verbal fluency in older adults.	*N* = 45. Older adults (25 women, 20 men. Range = 66.55, SD = 4.32). French Monolingual, sensory, cognitive and psychiatric immunity. All self-sufficient.	Word retrieval/lexical-phonological and inhibitory retrieval processes	Older people had to perform a verbal fluency task (words that begin with the letter “p” for 1 min). Then, they had to perform the verbal task of counting numbers out loud. Participants had to look at a black cross drawn in the center of a paper while performing the task. Pupil dilation was evaluated using Eye-Tracking glasses.	Larger pupil diameter was observed during the verbal fluency task (*M* = 2.57, SD = 1.03) than during counting (*M* = 2.03, SD =0.83) [t(45) = 2.84, *p* = 0.007, Cohen’s *d* = 0.58]. Also, participants generated a mean of 17.82 words (SD = 5.72) on the verbal fluency task, which is within the normal range.	The increased pupil size during verbal fluency can be attributed to the cognitive load of the task. Specifically, involves processes such as the retrieval of lexical information from the semantic knowledge-base, matching the retrieved information with the required category, initiating an articulatory plan, and, critically, inhibit and others. Pupillometry can be used as an ecological physiological assessment of verbal fluency in older adults.
El Haj et al. (2024)	Pupil size shows diminished increases on verbal fluency tasks in patients with behavioral-variant-frontotemporal dementia.	To investigated whether the difficulties in verbal fluency of patients with behavioral variant Fronto Temporal Dementia (bvFTD) can be assessed by measuring pupil size.	*N* = 24. 12 patients meeting criteria for the behavioral variant of frontotemporal dementia (bvFTD, 7 men and 5 women, M age = 66.21). 12 control participants (7 men and 5 women, M age = 65.12 years)	Word retrieval/lexical-phonological and inhibitory retrieval processes	Participants performed two verbal fluency tasks: letter fluency (letter p) and category fluency (animals) as well as a control task (counting aloud). The tasks were successively assessed but their order was counterbalanced.	Smaller pupil size was observed in patients with bvFTD compared to control participants on the letter fluency task (*p* = 0.001), category fluency task (*p* < 0.001), and the counting task (*p* = 0.034). As well demonstrated larger pupil size on the letter fluency task than on the counting task (*p* = 0.019), and category fluency task than on the counting task (*p* = 0.025).	While patients with dFTb show mainly personality and behavioral changes, language difficulties are also observed. The study provides an ecologically sound and reliable physiological assessment of language in bvFTD, paving the way toward the use of pupillometry in the cognitive assessment of patients with bvFTD.

El Haj et al. (2020) assessed pupillary response in a phonemic verbal fluency task (retrieval of multiple lexical items). Forty-five young adult participants were asked to retrieve all words beginning with the phoneme “p” and then to count numbers aloud while their pupillary dilation was tracked. As a result, greater pupillary dilation was obtained for the verbal fluency task compared to the counting task. The authors suggest that the increased pupil dilation during verbal fluency can be attributed to the cognitive requirement of the task, as participants were required to retrieve words, suppress irrelevant information, and explore alternative options. The same research team (El Haj et al., 2022) repeated the experiment with a group of older adults (*n* = 45). The results indicated that pupillary dilation increases among older adults during the verbal fluency task. For both studies, the researchers project that pupillometry could be helpful as a method of ecologically assessing lexical retrieval skills at different stages of the life cycle. In a recent publication, El Haj et al. (2024) evaluated individuals diagnosed with bvFTD compared to controls. Consequently, individuals with bvFTD showed less pupil dilation during the letter and category verbal fluency task (animals) compared to the control group. However, when comparing both tasks with counting numbers, the bvFTD group showed a greater pupillary response. The authors explore pupillometry’s applicability and ecological importance in investigating language processing in persons with dementia.

Finally, Ryals et al. (2021) explored how sensitive the pupillary response is during TOTs. They evaluated 46 undergraduates who were asked to answer pre-made questions. Participants had to report the sensation of TOTs when they could not retrieve the words associated with the requested questions. The results showed that TOTs generated a significant pupillary response (increase in diameter). The authors suggest that the increase in pupil size supports the notion that TOTs are linked to feelings of curiosity and the desire for knowledge, with the degree of pupil dilation corresponding to the amount of information retrieved. Therefore, pupillometry would be an appropriate and valuable tool for studying these linguistic phenomena.

## 4 Discussion

This section provides a general reflection and discussion of the findings presented in the results section. We begin with a discussion of the applicability, usefulness and purpose of pupillometry in lexical access studies. This is followed by a discussion of aspects related to the research methods and procedures used. Next, the sensitivity of this measure and the reliability of the results are discussed. Some basic recommendations for the conduct of lexical-pupillometric studies are then presented. Finally, some projections and limitations are briefly discussed.

### 4.1 Applicability, usefulness and purposes

The general interest in cognitive neuroscience in studying systematic changes in pupillary dilation is based on the fact that it is a noninvasive, objective and automatic neurophysiological measure (Hepach, 2023), which can reflect-if well controlled-the behavior of multiple cognitive constructs in diverse populations and life cycle stages (Laeng et al., 2012; Kafkas and Montaldi, 2015). In this context, the results of this review support that the pupillary response evoked by language processing tasks-and reflecting neuronal activity-has become an emerging area of development in experimental psycholinguistics over the last decade. However, its applicability is not exclusive to language processing. Our findings suggest that around 80% of the articles recovered during the identification phase were associated with various cognitive domains such as memory, attention, motivation, and emotions, where language tasks were merely one aspect of their data-gathering strategies and did not play a significant role. Out of the total, only 20% of the studies explored language processing, covering various levels (syntax, auditory recognition, speech perception, and lexical access) across different age groups and in neurotypical individuals or with neuro-cognitive disorders (congenital and acquired).

In particular, the applicability and usefulness of pupillometry as a method to assess cognitive processes have been demonstrated in several research fields (Ariel and Castel, 2014; Gomes et al., 2015; Kafkas and Montaldi, 2015; Larsen and Waters, 2018; de Winter et al., 2021). These areas comprise surprise and expectancy paradigms (Pätzold and Liszkowski, 2019), memory and attention tasks (Larsen and Waters, 2018; de Winter et al., 2021), or arousal and motivational states (Hepach et al., 2019). Regarding language and lexical access, we found that only 16 articles fully met the established criteria. Nonetheless, the careful examination of their methods, pupillometric analysis, statistical procedures, and outcomes provides sufficient evidence to assert that pupillometry is a versatile, valuable, and sensitive method for assessing linguistic processes involved in word recognition, retrieval, and semantic activation, and that it can be applied to any population, age group, and native or bilingual speakers. Indeed, the studies demonstrated that pupillary changes – measured mainly using peaks and dilation latencies – effectively reflect lexical activity, which allowed effects to be tested in 94% of the reports analyzed.

Furthermore, the present review demonstrates that the research purposes (objectives) in lexical access and pupillometry are also very diverse and varied, ranging from pupillary response in emotional word recognition in bilinguals (Toivo and Scheepers, 2019) to pupillometric behavior in lexical fluency tasks in individuals with bvFTD (El Haj et al., 2024). The same applies to the designs, techniques, and procedures applied, where pupillometry can be effectively used for word retrieval and semantic judgment tasks, as well as LDT (or its variations). In addition, there are differences in the cleaning, analysis, and interpretation of the pupil data. These variations include using different analysis windows, the basal pupil diameter used, the pupil variable examined (maximum pupil dilation vs. maximum latency), and other factors. Thus, the emerging development and the diversity of methods observed in this field, which can be considered an advantage, do not yet allow definitive and solid conclusions to be drawn in this area.

On the other hand, although the collected background information demonstrates the usefulness of pupillometry in addressing various research problems in lexical access, it would be incorrect to assume that any pupillary response indicates lexical activation. From a methodological point of view, it is always necessary to “isolate” other cognitive factors associated with pupillary dilation, such as surprise, attention, or task-associated motivation (Hepach, 2023), as they could mask the expected pupillary response. Hence, while pupillometry is undeniably useful for studying lexical access and shows promising growth potential, it still requires improved precision and standardized conditions of use that demonstrate that it truly represents the pupillary response obtained, as we are not entirely sure whether it is an exclusive response to the cognitive effort expended in the task (Van der Wel and Van Steenbergen, 2018), or a response to the attentional component present in the cognitive function performed (Strauch et al., 2022), or simply a specific response for language processing.”

In sum, this review validates the applicability of pupillometry as a sensitive and valuable technique for assessing various lexical levels, which is a positive attribute of this neurophysiological measure. However, due to the varied purposes, objectives, and data analysis approaches of the reviewed studies, we cannot establish definitive conclusions in this area. Multiple reasons could explain this: (1) Pupillometry is a recently popularized and recognized technique; (2) pupillary dilation is a very volatile signal whose control and instrumental measurement have recently achieved better management (Hepach, 2023); (3) the interest in its application and use is limited to specific research groups rather than large collaborative networks, who turned to pupillary dilation measurement as a practical and conceptual solution to their research problem (Schmidtke, 2018). In this sense, defining more standardized purposes for applying pupillometry in lexical access would enable us to produce far more accurate research purposes, enhancing the vigor and robustness of this study area.

### 4.2 Methods and procedures applied

There is a consensus that most studies in experimental psycholinguistics use small sample sizes, which contrasts with the large number of experimental trials used to favor greater statistical power (Perea and Rosa, 1999). In addition, these studies often include young, normotypical participants selected for interest, with the aim of testing hypotheses in a ‘healthy’ brain (Rojas et al., 2022). In this respect, lexical-pupillometric studies are no exception. For example, independent of the level of processing assessed, all articles exhibited bounded sample sizes in each experiment performed (i.e., Recognition: *n* = 35, Guasch et al., 2017; *n* = 96, Toivo and Scheepers, 2019; *n* = 68, Shechter et al., 2022. Semantic activation: *n* = 20, Egan et al., 2020; *n* = 30, Mathôt et al., 2017; *n* = 60, Geller et al., 2019. Retrieval: *n* = 45, El Haj et al., 2020, 2022; *n* = 46, Ryals et al., 2021), but they used a large number of experimental trials. Moreover, like the “more traditional” studies, the evaluated populations were preferably young university students with normal cognition, vision, and hearing. This situation varied when the object of study involved specific age groups or clinical conditions (*n* = 19 persons with dyslexia, Egan et al., 2023; *n* = 27 children under 24 months, Angulo-Chavira and Arias-Trejo, 2021; *n* = 24 patients with bvFTD, El Haj et al., 2024). Given the benefits of pupillometry (i.e., a non-invasive, objective, and free of conscious responses), sample sizes and types of participants observed in the studies reviewed, it is possible to assume that this technique could be applied in any population, particularly to individuals with complex motor, behavioral, or cognitive control, such as very young children (Hepach et al., 2019), older people and individuals with neurodegenerative diseases (El Haj et al., 2022, 2024). This fact reveals another advantage and endorses the potential of this neurophysiological tool.

Regarding the experimental tasks used in lexical-pupillometric studies, at the level of word recognition and similar to traditional studies in the field, two articles used classical LDTs (Guasch et al., 2017; Haro et al., 2017), and two additional studies used word recognition and subsequent reading methods (Shechter and Share, 2021; Shechter et al., 2022). On the other hand, regarding the activation and semantic processing level, only semantic judgment tasks were stated in several articles (Geller et al., 2019; Egan et al., 2020; Reilly et al., 2020). At the retrieval level, verbal fluency (El Haj et al., 2020, 2022, 2024) and naming by definitions (Ryals et al., 2021) tasks were preferentially used. Therefore, the assessment paradigms in the reviewed lexical-pupillometric studies are the same as the traditional ones. Additionally, they share similarities in the number of items used, the organization of each trial, the number of blocks, the stimulus presentation latencies, and the recorded behavioral responses (RT and accuracy). Similarly, lexical variables like frequency (high/low), lexicality (words/pseudowords), syllable length, polysemy, phonological form, and prime type, among others, are also subject to control. In this sense, pupillometry’s easy adaption to conventional research paradigms in experimental psycholinguistics supports its numerous uses and provides further evidence for its effectiveness in examining lexical access.

The situation becomes more confusing when analyzing and comparing the methods used to collect, control, and analyze the resulting eye data. Although, it should be recognized that basic points are consistent across articles: (1) pupillary data were recorded before, during, and after the presentation of the target stimulus; (2) areas of interest were previously identified according to the purpose of each examination, and (3) two classes of pupil measurements were preferably calculated: maximum dilation and maximum pupil latency (this is consistent with the language processing and pupillometry studies referenced by Schmidtke, 2018); it should also be noted that there are other aspects that need to be described and addressed in more detail (see point 4.4. Recommendations). In conclusion, although the necessary details for pupillometric control and analysis are reported, there are also some methodological omissions, probably made but not explicitly stated, which are necessary to replicate the studies presented here.

Hepach’s work (Hepach, 2023) defines some requirements for more effective pupillary dilation control at the experimental level. In this sense, the pupil response would not only reflect a particular cognitive response, but would also be the result of: (1) light adaptation to the environment and the focused visual field, (2) eye and head movements (Brisson et al., 2013), (3) changes in tonic arousal state (cardiac or cutaneous arousal state; Anderson and Colombo, 2009), and (4) the cognitive state of the participants (Eckstein et al., 2017). In this context, only a small number of the reviewed articles offer partial details regarding the light control of the environment, the particular visual field of focus, the participants’ head posture, and their state of arousal; except for the cognitive state, which was always stated and controlled. The lack of control of these experimental conditions and, in particular, the light verification during the application of the experiments (Schmidtke, 2018) are probably some of the main weaknesses observed in the reviewed studies.

### 4.3 Sensitivity of pupillometry and reliability of results

As mentioned, determining *a priori* that pupil size increase reflects only lexical activity (or word processing) may be risky and highly inaccurate. In contrast, pupillary responses may simultaneously encompass other cognitive processes (i.e., cognitive load, surprise, motivation; Larsen and Waters, 2018; Hepach et al., 2019; Pätzold and Liszkowski, 2019) and depend on external elements such as ambient illumination and intrinsic characteristics to the participant (Brisson et al., 2013; Eckstein et al., 2017). Therefore, the sensitivity and reliability of pupillometry will depend to a large extent on methodologically rigorous and specific experiments and their subsequent analysis. In this regard, all the articles reviewed proved to be very strict both in the approach to the lexical task and in the subsequent pupillometric analysis (although, as already described, in some cases, specific methodological parameters necessary for replication were not explicitly stated). Therefore, apart from some observed weaknesses, the analysis allows us to argue that the pupillometric results obtained seem to represent, in whole or in part, the lexical processing of words.

At the statistical level, the analyzed lexical-pupillometric results (i.e., maximum pupil dilation or maximum pupil latency) are quite clear and significant at the different levels tested. For example, in word recognition tasks, statistically significant (*p* < 0.05) pupillary dilations were evidenced when recognizing low-frequency words (Haro et al., 2017), words without cognate status (Guasch et al., 2017), emotional words in L1 (Toivo and Scheepers, 2019) and when processing pseudowords (Shechter and Share, 2021). Concerning semantic activation, significant pupillary dilation has been observed in the processing of words with low semantic relatedness (Geller et al., 2019), in the activation of taboo words (post-transcranial stimulation, Reilly et al., 2020), and in the processing of words that are semantically related or consistent to a semantic field (Egan et al., 2020; Haro et al., 2023). At retrieval level, significant pupillary dilation (*p* < 0.05) occurs when performing verbal fluency tasks and when TOTs are present (El Haj et al., 2020, 2022, 2024; Ryals et al., 2021). In sum, although the number of articles reviewed is relatively small to determine exactly how suitable pupillometry is for the study of word processing, it is striking that 94% of the articles reviewed showed the expected effects, which seems to support the idea that the technique does indeed appear to be sensitive enough for the study of lexical processing.

However, the same cannot be said for the reliability of pupillometric measures. With the evidence reviewed in the present review, it cannot yet be determined whether pupil dilation is specifically and uniquely related to language processing. It is also uncertain whether the pupil dilation levels obtained in the applied tasks are not at least partially influenced by other cognitive processes, such as motivation, arousal, and attention, as evidence from other fields shows (Hepach et al., 2019; Pätzold and Liszkowski, 2019). In this sense, the influence of other cognitive processes on pupillometric measures is not in itself a problem; many of the linguistic processes are not exclusively linguistic and a large number of factors are involved in both the comprehension and production of language. However, it is necessary to determine the relative weight of each factor in the pupillometric measures obtained in order to better isolate specific aspects of language processing.

In this context, we hypothesize that the different neuromodulatory systems responsible for pupil activity (PON/SC/LC, Joshi and Gold, 2020; Joshi, 2021) operate as interconnected systems that generate specific pupil responses in the presence of different cognitive functions (i.e., attention, language, emotion, working memory). Therefore, we think word processing (or lexical access) may generate pupil dilation patterns specific to this function (i.e., reflected in specific temporal dilation window, mean dilation diameters or particular dilation peaks). Thus, it is likely that there are distinct dilation components for linguistic tasks related to word recognition, lexical retrieval, and semantic activation. However, these specific pupil dilation components need to be isolated and distinguished from other response components (similar to the behavior of Evoked response potential/ERP components), such as cognitive effort, surprise, attention, or memory, which are likely to manifest themselves through pupil response patterns that are different and distinct from the ‘linguistic’ pupil response. Thus, determining which cognitive-neural processes are actually represented by changes in pupil size has become one of the major current challenges for cognitive neuroscience.

One way to ensure the reliability of lexical-pupillometric results is for studies using this measure to generate their own experimental designs. In other words, these experimental designs should be intended as pupillometric studies from the initial stage of the experimental design and not at later stages (Hepach, 2023). Hepach (2023) mentions that it would not be advisable to ‘salvage’ data from a completed (and for other purposes) eye movement study to perform a supplementary or secondary pupillometric analysis, as there are numerous artifacts and experimental manipulations that could alter the results. Consequently, any study examining pupillary changes during word retrieval, semantic activation, or recognition should be designed from the beginning as a pupillometric study. This includes considerations for sample size, basal pupil diameter definition, analysis window duration, control over blink frequency, saccades, and other artifacts, control over illumination (i.e., matching the luminance of the stimuli or using the same image in all experimental conditions), excitability and cognitive characteristics of the persons assessed, among other aspects.

### 4.4 Basic recommendations for conducting lexical-pupillometric studies

The results of the present review allow us to propose some basic recommendations for using pupillometry in lexical access tasks. First of all, considering the multiple applications of this neurophysiological measure, its different forms of measurement and the different alternatives in data analysis, we suggest that, before formulating the experiments, the researcher should: (1) deepen his or her physiological knowledge of the pupillary response; (2) inquire about technical and procedural aspects already established in lexical-pupillometric experiments; and (3) formulate or replicate objectives based on previous studies in the field, which will allow improving the consistency and stability of research purpose in different populations and contexts.

Secondly, we have found certain methodological and procedural weaknesses in several lexical-pupillometric studies that should be described and addressed in more detail. For example, more precise control over the number of blinks, saccadic movements, and other artifacts is recommended. It is known that data ‘contaminated’ by blinks (*N* > 5) and saccades (*N* > 5) in five or more trials throughout the experiment can lead to erroneous results (Kafkas and Montaldi, 2015; Carbajal et al., 2018; McKee et al., 2018). Similarly, the differences in measurement units used for pupil dilation (arbitrary chamber units vs. metric units) and the lack of explanation for choosing one measurement over another must be addressed. It is also recommended to comprehensively describe the methodology used to calculate the mean number of eye fixations and the mean fixation duration for each trial. In addition, it is recommended to provide further explanation on how the basal pupil diameter was calculated: by averaging the anterior pupil diameter during the target appearance or while the fixation cross was displayed? In this sense, for future research in this area, we propose to review the methodological considerations for using pupillometry in the study of language processing, according to Schmidtke (2018).

Thirdly, there is an urgent need to replicate already published studies, increase sample sizes and standardize specific parameters of pupillary analysis in order to increase the reliability of this neurophysiological measurement (Hepach, 2023). Thus, greater and better replicability of studies and standardization of the forms of recording, administration and analysis of pupillometric results will favor the knowledge and reliability of the procedure and allow access to more consistent language processing data.

Finally, as general recommendations, Mathôt and Vilotijević (2023) proposed a practical guide for the general design of cognitive experiments using pupillometry. In their paper, they propose six basic principles for experimental design: (1) stimuli used in experiments should be constant across conditions; (2) eye position should also be constant across conditions; (3) tests should be presented at a slow presentation rate; (4) pupil size should be assessed while participants are doing nothing; (5) ambient lighting should be intermediate and adapted to the brightness of the screen; and (6) data should be stored in a single file per participant (for further details see Mathôt and Vilotijević, 2023). In addition, for the correct selection of participants, recording characteristics, stimulus and timing parameters, data accessory noise, measurement procedures, and presentation of results and plots, we suggest reviewing the paper by Steinhauer et al. (2022), “*Publication guidelines and recommendations for pupillary measurement in psychophysiological studies*.” Finally, we recommend reviewing the paper by Kret and Sjak-Shie (2019), “*Preprocessing pupil size data: Guidelines and code*,” for guidelines and technical suggestions for correct pupil data processing and statistical analysis.

### 4.5 Projections and limitations

The projections of pupillometry in the study of lexical access are invaluable in developing a better understanding of how people process information. First, it is an objective, non-invasive neurophysiological tool without voluntary response mechanisms (Haro et al., 2017), which provides information about the time course of the process being assessed (Guasch et al., 2017). Second, its high adaptability and usefulness are favorable for studying word recognition in different age groups, both in the native language and in L2 for bilinguals (Toivo and Scheepers, 2019), and it can be coupled with LDT or recognition-reading tasks. In addition, it allows working on semantic activation tasks, such as word retrieval, in neurotypical individuals or those with complex motor, cognitive, and behavioral control (Angulo-Chavira and Arias-Trejo, 2021; El Haj et al., 2024). Third, while specific methodological issues with pupillometric data analysis can be fixed, the reviewed studies demonstrate a high degree of rigor and thoroughness in their procedures, leading to results that seem sensitive.

Furthermore, the technology used to record and measure pupillary behavior is expected to advance in accuracy and dependability, given its volatility (Hepach, 2023). Enhancing these parameters will make it possible to record responses in greater detail, making it easier to interpret the anticipated effects. Moreover, a more systematic integration of lexical and pupillometry research with other neuroimaging technologies is necessary. Combining various measurement modalities can yield a more thorough understanding of the linguistic-cognitive processes under examination. This would not only help to increase knowledge in a normotypical population, but could also strengthen clinical research into the understanding and diagnosis of neuropsychiatric disorders, as well as the development of future biomarkers for the early detection of Alzheimer’s, Parkinson’s, autism spectrum disorders and other neuropsychiatric disorders.

Pupillometry also has limitations that have partially impeded its advancement in cognitive neuroscience. The first and perhaps most common reason is that several factors affect pupil dilation, such as ambient lighting, age, fatigue, and the person’s general state of alertness, making it an extremely volatile measure that is difficult to capture and interpret. This limitation is compounded by non-cognitive autonomic physiological perturbations (stress response or direct light stimulation of the experimental material), where distinguishing between cognitive and non-cognitive responses is challenging. An additional constraint pertains to the inter-individual variability in pupillary response (basal pupil diameter) among participants. This variability must be considered when analyzing the data since a typical response for one participant might not be typical for another.

Finally, on a technical level, pupillometry requires a thorough knowledge and handling of the software and hardware used, which is essential for reliable measurements. Inappropriate pupil calibration or reading may result in errors in the interpretation of results. A reliable (and therefore reproducible) pupillary response requires strict and controlled experimental and methodological conditions. A “light” or loose experimental design may influence pupillary response or reflect other cognitive processes. Thus, despite some limitations, pupillometry is a valuable tool in cognitive research and, in this particular case, in the study of lexical processing. By combining pupillometry results with those obtained from other measures and techniques, researchers can gain a more complete understanding of the cognitive processes under study. Of course, it is important to recognize and address these limitations in order to generate and interpret these data reliably and accurately.

## 5 Conclusion

In this scoping review, we have attempted to determine the applicability and usefulness of pupillometry in the study of lexical access and, for the first time, provide an updated overview of research in this specific field of study. The results demonstrate that pupillometry is a highly applicable and useful method for assessing various word recognition, retrieval, and semantic activation skills, that easily fits and complements traditional lexical access research paradigms and methods. In addition, the strong methodological rigor of its application gives it a good level of sensitivity to the results obtained. Considering that pupillometry is a non-invasive, objective procedure without conscious reactions, it could be a technique applicable to any population and age group, especially those with complex motor, behavioral, or cognitive control.

In turn, we think that word processing (or lexical access) may generate pupil dilation patterns specific (i.e., temporal dilation window, mean dilation diameter, or dilation peaks) for this function, which should be isolated and distinguished from other response components such as cognitive effort, surprise, attention or memory. However, the emerging development of this specific area of research and the methodological diversity observed in the included studies (which can be considered an advantage to some extent due to its versatility) do not yet allow for definitive and robust conclusions in this area, which in turn does not allow for meta-analyses or entirely conclusive statements about what the pupil response actually reflects when processing words. Defining and detailing standardized methods for recording and analyzing pupillary data in lexical access tasks would enable the creation of more accurate, reliable, and reproducible research designs and add more power and robustness to this line of research. Finally, lexical access research using pupillometric methods currently holds incalculable potential, so better knowledge and standardization of this procedure - and its integration with other technologies - would not only contribute to increasing knowledge about language processing in neurotypical populations across the lifespan but also could strengthen clinical research to understand linguistic-cognitive functions in various neuropsychiatric disorders.

## Data availability statement

The raw data supporting the conclusions of this article will be made available by the authors, without undue reservation.

## Author contributions

CR: Conceptualization, Data curation, Formal analysis, Funding acquisition, Investigation, Methodology, Project administration, Resources, Supervision, Validation, Writing – original draft, Writing – review & editing. YV-R: Conceptualization, Formal analysis, Investigation, Visualization, Writing – original draft, Writing – review & editing. GL: Conceptualization, Investigation, Methodology, Supervision, Visualization, Writing – original draft. MC-M: Conceptualization, Data curation, Formal analysis, Investigation, Methodology, Visualization, Writing – original draft, Writing – review & editing. YS: Conceptualization, Investigation, Software, Writing – original draft, Writing – review & editing. JC-A: Conceptualization, Data curation, Formal analysis, Investigation, Software, Supervision, Validation, Visualization, Writing – original draft, Writing – review & editing.

## Footnotes

^1^ https://rayyan.ai/
